# Rumen microbial communities influence metabolic phenotypes in lambs

**DOI:** 10.3389/fmicb.2015.01060

**Published:** 2015-10-12

**Authors:** Diego P. Morgavi, Estelle Rathahao-Paris, Milka Popova, Julien Boccard, Kristian F. Nielsen, Hamid Boudra

**Affiliations:** ^1^Institute National de la Recherche Agronomique, UMR1213 Herbivores, Clermont Université, VetAgro Sup, UMR HerbivoresClermont-Ferrand, France; ^2^Institute National de la Recherche Agronomique, UMR 1145 Ingénierie Procédés AlimentsParis, France; ^3^Agroparistech, UMR 1145 Ingénierie Procédés AlimentsParis, France; ^4^School of Pharmaceutical Sciences, University of Geneva, University of LausanneGeneva, Switzerland; ^5^Department of Systems Biology, Technical University of DenmarkKgs. Lyngby, Denmark

**Keywords:** gut microbial colonization, host-microbe interaction, urine metabolome, rumen archaea, rumen bacteria, rumen protozoa

## Abstract

The rumen microbiota is an essential part of ruminants shaping their nutrition and health. Despite its importance, it is not fully understood how various groups of rumen microbes affect host-microbe relationships and functions. The aim of the study was to simultaneously explore the rumen microbiota and the metabolic phenotype of lambs for identifying host-microbe associations and potential biomarkers of digestive functions. Twin lambs, separated in two groups after birth were exposed to practices (isolation and gavage with rumen fluid with protozoa or protozoa-depleted) that differentially restricted the acquisition of microbes. Rumen microbiota, fermentation parameters, digestibility and growth were monitored for up to 31 weeks of age. Microbiota assembled in isolation from other ruminants lacked protozoa and had low bacterial and archaeal diversity whereas digestibility was not affected. Exposure to adult sheep microbiota increased bacterial and archaeal diversity independently of protozoa presence. For archaea, Methanomassiliicoccales displaced Methanosphaera. Notwithstanding, protozoa induced differences in functional traits such as digestibility and significantly shaped bacterial community structure, notably *Ruminococcaceae* and *Lachnospiraceae* lower up to 6 folds, *Prevotellaceae* lower by ~40%, and *Clostridiaceae* and *Veillonellaceae* higher up to 10 folds compared to microbiota without protozoa. An orthogonal partial least squares-discriminant analysis of urinary metabolome matched differences in microbiota structure. Discriminant metabolites were mainly involved in amino acids and protein metabolic pathways while a negative interaction was observed between methylotrophic methanogens Methanomassiliicoccales and trimethylamine N-oxide. These results stress the influence of gut microbes on animal phenotype and show the potential of metabolomics for monitoring rumen microbial functions.

## Introduction

The gut microbiota of higher animals provides a myriad of ecosystem services to the host-microbe holobiont. In the case of livestock animals, these ecosystem services provide food and income to hundreds of millions of people worldwide (Raney et al., 2009). Among livestock species, ruminants in particular have evolved an exquisite foregut fermentation system (rumen or reticulo-rumen) that harbors symbiotic microbes capable of harvesting energy from otherwise indigestible structural plant polysaccharides not suitable for human consumption. The functional plasticity of the rumen microbiota makes ruminants highly adaptable to different diets (Morgavi et al., 2013). However, the rumen microbiota is also responsible for the production of the greenhouse gas methane and nitrogen-rich wastes (Martin et al., 2010; Morgavi et al., 2010). A recent global assessment of livestock systems identified feed efficiency, a trait closely associated to the microbiota, as the key driver to improve productivity and reduce emissions (Herrero et al., 2013). These data point out that a better understanding of the gut microbial community and its interactions with the host are necessary for the sustainability of ruminant production systems.

An essential process for the proper functioning of the gastrointestinal microbiota is the recruitment and development of their various microbial denizens (Costello et al., 2012). There are multiple elements that govern the establishment of a functional gut microbiota in young animals. The assembly of different microbial phylotypes starts from birth and is influenced by diet and the immediate environment, notably the mother and contact with other congeners. The anatomical development of the gastrointestinal tract and the luminal environment also play a major influencing role in the acquisition of a stable microbiota. Ruminants are colonized by bacteria and methanogenic archaea in the first days of life (Fonty et al., 1987; Skillman et al., 2004; Jami et al., 2013), whereas different anaerobic fungal and protozoal populations became established between 2 and 8 weeks of age (Fonty et al., 1987, 1988). Although the composition of rumen bacterial communities using high throughput sequencing approaches have been reported in young ruminants (Li et al., 2012; Jami et al., 2013; Rey et al., 2014), there is still a lack of information on other important microbial groups and, more importantly, the influence of microbiota composition on the host metabolome remains largely unknown.

In this work, we used young ruminants subjected to different microbial-modulating interventions to simultaneously explore the rumen bacterial and archaeal communities, the urine metabolome and other general traits attributed to the capacity of the microbiota to utilize feeds such as digestibility, ingestion and live weight gain. This study brings new insights on the interactions between gut microbial populations among themselves and with the host and highlights the importance of rumen microbes as modifiers of the host metabolic phenotype.

## Materials and methods

Procedures with animals were conducted in accordance with the guidelines for animal research of the French Ministry of Agriculture and applicable European guidelines and regulations for experimentation with animals. Four pairs of twins INRA 401 male lambs born within a week interval were used in this study. Lambs were monitored for colostrum sucking and were separated from their mothers at about 12 h after birth. Lambs were separated in two groups with one twin brother per group. Each group was housed in separate pens, fed with milk replacer and had access to clean water and a standard diet of hay and concentrate. The groups were not in direct contact and did not have any contact with other ruminants but otherwise they were housed under normal rearing conditions. Milk feeding was stopped at the age of 9 weeks. The trial lasted 31 weeks and consisted of four digestibility and sampling periods. Lambs were fed once a day a fixed amount of concentrate (800 g, crude protein 14%, starch 65%) that was constant throughout the trial and hay was fed at libitum. In period 1, sampling and individual digestibility measures were performed in week 14. At the age of 15 weeks, lambs in group 1 (G1) were inoculated with 100 ml rumen fluid obtained from two rumen-cannulated wethers containing a conventional microbiota. Donor wethers were fed a hay diet once a day. Rumen contents were taken before feeding, strained through a polyester monofilament fabric (250 μm mesh aperture) and pooled to produce the inoculum. The concentration of protozoa was 5.3 log_10_/ml.

The second group (G2) was inoculated 1 week later with the same inoculum (100 ml/lamb) that was kept frozen as a way to induce changes in the microbial community. Protozoa in particular are labile to freeze-thawing (Nsabimana et al., 2003) and we used this process to produce a “stressed” inoculum for G2. Six weeks after inoculation (20 weeks of age) sampling and individual digestibility measurements were done as for period 1. From 22 to 26 weeks of age lambs in both groups were administered a mild toxin, ochratoxin A (OTA) dosed once a day at 30 μg OTA/kg BW before the feeding in the morning. On the last week of dosing, sampling and individual digestibility measures were done as before. At the dose used, OTA is not toxic to rumen microbes and to the animal. We hypothesized that long term exposure to OTA might affect microbiota composition and urine metabolome. This toxin might be degraded at different rates in the rumen by different microbial groups, being protozoa particularly active (Mobashar et al., 2012). The toxicokinetics of OTA will be reported elsewhere. In the last period, lambs in both groups were gathered in a common pen. Sampling and digestibility measurements were performed 5 weeks after, at 31 weeks of age. Lambs were weighed regularly during the study.

Digestibility procedures, urine and rumen fluid sampling for analysis were carried out following standard procedures (Supplementary Material). Feeding and any manipulation on lambs was done in the morning before handlers contacted other animals. It started always with G2 and included a change of protective clothing before tending to G1. Bed cleaning was done with minimal contact by grouping the lambs in one corner of the pen.

### Urinary fingerprint analysis (metabolome)

Liquid chromatography (LC) separation was performed by reversed phase chromatography on a 10 cm Luna C18(2) columns using an acidic water-acetonitrile system as described in detail in the Supplementary Material. The LC was interfaced to a Micromass LCT Time of flight MS instrument using an ESI interface and running in positive mode scanning m/z 100–900 (Nielsen et al., 2011).

### Amplicon sequencing and processing

Total gDNA was extracted using the method described by Yu and Morrison (2004) and quantified by spectrophotometry using a NanoQuant Plate on an Infinity spectrophotometer (TECAN, Switzerland). Amplicon sequencing of ruminal gDNA samples was done by MR DNA (Shallowater, TX, USA). For bacteria, amplification was performed using primers F515/R806 targeting the V4 region of the 16S rRNA gene (Caporaso et al., 2011). For archaea, primers Arch349F and Arch806R were used (Takai and Horikoshi, 2000). PCR was performed using HotStarTaq Plus Master Mix Kit (Qiagen, USA) on the following conditions: a starting cycle at 94°C for 3 min, followed by 28 cycles at 94°C for 30 s, 53°C for 40 s and 72°C for 1 min, and a final cycle with an elongation step at 72°C for 5 min. Sequencing was performed on an Ion Torrent PGM following the manufacturer's guidelines for bacterial amplicons and on a Roche 454 FLX titanium instrument for archaeal amplicons (Dowd et al., 2008).

Approximately 2 × 10^6^ raw bacterial reads were produced from bacterial primers. Reads were assigned to their respective rumen samples and trimmed of forward and reverse primers. Minimum sequence length was set at 150 bp. Operational taxonomic units (OTUs) were clustered at 97% of similarity and filtered using USEARCH (Edgar, 2010). Following taxonomy assignment, non-bacterial sequences were removed. Alignment was done using PyNAST (Caporaso et al., 2010a) and taxonomy with the RDP classifier (Wang et al., 2007) against the Greengenes database (gg_otus-12_10-release).

Raw archaeal reads were analyzed as bacterial reads but the minimum sequence length was set at 200 bp and following taxonomy assignment non-archaeal sequences were removed. A total of 193,367 raw reads were obtained from the pyrosequencing platform using the archaeal primers.

The sequence data have been submitted to the EMBL databases under accession No PRJEB8814. Raw sequences were filtered and analyzed using the QIIME v. 1.7.0 pipeline with default options unless otherwise stated (Caporaso et al., 2010b). Chao1 for calculating richness and Shannon that accounts for abundance and evenness were calculated using QIIME. As an additional estimator of the representativeness of the sequences obtained, Good's coverage was calculated as G = 1−n/N, where n is the number of singleton OTUs and N is the total number of sequences in the sample. Rarefaction analysis was done using the lowest number of reads obtained from a sample for each of the target communities and a cut-off of 97%.

Other microbial analysis. Protozoa enumeration was done using a Neubauer counting chamber. Total bacteria, *Fibrobacter succinogenes, Ruminococcus albus, R. flavefaciens*, and *Selenomonas ruminantium* were evaluated by quantitative (q)PCR using primers targeting the 16S rRNA gene (Edwards et al., 2007; Stevenson and Weimer, 2007; Mosoni et al., 2011, Supplementary Material).

### Statistical analysis

Rumen fermentation parameters, intake, digestibility, and body weight data were analyzed in repeated measures using the MIXED procedure of SAS v9 (SAS Institute Inc., Cary, NC). The model included the fixed effect of inoculum, lambs age, group, and period × group interaction. The animal was considered as a random effect. Best fitting covariance structure was compound symmetry. Differences were tested with the LSMEANS statement for period, group and their interaction and with the LSMESTIMATE statement for testing the effect of inoculum at 20 and 26 weeks of age. Significance was declared at *P* < 0.05 probability level and trends were discussed at *P* < 0.10 probability level.

The Kruskal-Wallis test as implemented in SAS was used to estimate the difference between samples in the number of sequenced reads. Differences in the abundance of reads attributed to phyla, families and genera were done using Metastats (White et al., 2009).

MS metabolomic data were first processed using XCMS (Smith et al., 2006) running under R version 2.11.1, generating a table of mass and retention time with associated signal intensities for all detected peaks. Data were normalized to the sum of all ions intensity of each sample. Each variable was then standardized using the square root of its standard deviation as scaling factor, i.e., Pareto scaling, and the resulting data matrix was further analyzed using multivariate methods to uncover trends of metabolic patterns. Data exploration was done using unsupervised and supervised methods: principal component analysis (PCA) and orthogonal partial least-squares discriminant-analysis (OPLS-DA), respectively. All single-block models were computed with the SIMCA-P software (Umetrics, v. 13.03, Sweden). Leave-one-out cross-validation was used to evaluate optimal model size based on goodness of prediction Q^2^. Model validity was verified using permutation tests. For each OPLS-DA model, the most discriminating variables were highlighted based on variable importance in the projection (VIP) and S-plots. The significance of individual variables between groups (G1 vs. G2) was further assessed using ANOVA test (R software, version 2.11.1). Consensus OPLS-DA was used to combine metabolomics and microbial data (Boccard and Rutledge, 2013).

## Results

The current study assessed the impact of different microbial-modulating events on rumen microbiota and metabolic phenotype in lambs. We used lambs as animal model because the precocial characteristic of the species allows the separation of lambs from their mothers soon after birth and makes it possible to control the diet and the surrounding environment. In addition, the possibility to have twins that were allocated to different groups reduced possible confounding maternal effects. At the time of the first measurements, lambs had been weaned for 1 month, had a functional rumen (Wardrop and Coombe, 1961) and consumed the same diet that was fed throughout the experiment thus minimizing additional confounding factors.

Throughout the trial, none of the lambs showed signs of health or behavioral problems. The “stressed” freeze-thawed rumen inoculum effectively killed most protozoal cells. At 20 weeks, only one lamb in G2 had *Ophryoscolecidae* protozoa at relatively low concentrations and at weeks 26 *Ophryoscolecidae* were present in a second lamb. In contrast, representatives of the *Isotrichidae* were not present in G2. For G1, all lambs had concentrations comparable to conventionally reared sheep composed of both *Ophryoscolecidae* and *Isotrichidae*. The values were stable throughout the experimental period with average concentrations of 6.2 log_10_/ml rumen fluid (Supplementary Figure S1).

### Rumen bacteria

A total of 905 979 bacterial reads were retained following filtering to exclude low quality reads, chimeras and low-presence OTUs up to doubletons. The average per sample was 28,312 ± 8543 with no differences revealed between the two groups and the four samplings at different ages (*P* = 0.15, *n* = 4). The Good's coverage estimator returned the highest coverage values for 14 weeks lambs at 99.3%. As lambs aged, the coverage value decreased in subsequent periods. However, the coverage at the end of the 31-week experiment was still good at =97.8% meaning that over 45 reads were needed to detect a new phylotype. Accordingly, rarefactions curves for younger animals started to flatten earlier than for older animals (Supplementary Figure S2A). The Shannon index shows the lowest diversity for 14-week-old lambs in G2 (Table 1). In contrast, smaller differences were detected between other samplings either between groups or through time.

**Table 1:** **Shannon diversity index of rumen bacteria and archaea in lambs as a function of interventions modulating the rumen microbiota^a^**.

**Shannon index**	**Group 1**				**Group 2**				**SEM**
	**Fauna-free 14 week**	**Faunated 20 week**	**Faunated 26 week**	**Faunated 31 week**	**Fauna-free 14 week**	**Fauna-free 20 week**	**Fauna-free 26 week**	**Faunated 31 week**	
Bacteria	7.2^AB^	7.7^AB^	8.0^A^	8.0^A^	6.3^C^	7.0^BC^	7.1^AB^	7.9^A^	0.59
Archaea	2.6^BC^	2.9^AB^	3.3^AB^	3.5^A^	2.0^DC^	1.7^D^	2.6^BC^	3.5^A^	0.51

a At 15 weeks of age, lambs were gavaged with fresh (group 1) or freeze-thaw (group 2) rumen microbial inocula from adult sheep. From week 21 through 26 lambs received daily a mild dose of ochratoxin A, and from week 27 lambs were in a common pen. The presence and abundance of protozoa influenced the results and the terms fauna-free and faunated are used to simplify the presentation. For each row means with different superscript letters differ (P < 0.05; n = 4).

The main phyla detected in all samples were the Bacteroidetes and Firmicutes as expected for gut-associated bacterial communities in mammals. These two phyla represented on average 52 and 30% of the sequences, respectively. Another prominent phylum was Fibrobacteres, which was absent in 14-week-old lambs but, following inoculation with rumen fluid from adult animals, represented on average 13% of sequences in both 20-week-old lambs and in older lambs. Proteobacteria accounted for ~3% of sequences but their proportion in samples varied between animals and periods, i.e., the large proportion seen in G2 at 14 weeks was driven by a single animal that had up to 30% of total counts represented by Proteobacteria (Table 2; Supplementary Figure S3). As lambs aged, individual differences were less pronounced.

**Table 2:** **Relative abundance of rumen bacterial phyla in lambs as a function of interventions modulating the rumen microbiota^a^**.

**Phylum**	**Group**	**Age (week)**												**Comparison within group**		
		**14**			**20**			**26**			**31**			**14 vs. 20 week**	**20 vs. 26 week**	**26 vs. 31 week**
		**mean**	**SE**	***P***	**mean**	**SE**	***P***	**mean**	**SE**	***P***	**mean**	**SE**	***P***			
Bacteroidetes	1	54.57	1.66		42.54	4.88		54.25	1.54		53.03	3.84		^*^	^*^	
	2	54.69	4.27		43.72	4.05		59.45	3.67		54.11	3.82		*0.07*	^*^	
Firmicutes	1	41.07	1.89		35.37	2.16		33.10	2.56	^**^	20.49	0.78		*0.06*		^**^
	2	31.82	8.52		28.80	6.53		23.44	1.97		23.37	1.75				
Fibrobacteres	1				17.50	3.86		2.19	0.61	^**^	13.60	1.73		^**^	^**^	^**^
	2				18.60	8.97		12.29	3.25		11.43	3.23		^*^		
Proteobacteria	1	1.21	0.84		0.99	0.55		0.87	0.33		1.99	1.03				
	2	9.28	7.90		5.83	3.33		2.07	0.91		1.05	0.16				
Tenericutes	1	0.72	0.32		0.75	0.20		1.77	0.49	^*^	1.00	0.10			*0.06*	
	2	0.62	0.41		0.92	0.23		0.37	0.11		3.01	1.49			^*^	
Synergistetes	1	0.11	0.03		0.17	0.05		1.54	0.86		2.66	1.00				
	2	0.05	0.02		0.07	0.03		0.17	0.06		0.79	0.65				
Actinobacteria	1	0.47	0.16	*0.06*	0.09	0.07		0.06	0.02	*0.07*	0.03	0.01	^*^	^*^		
	2	2.78	1.24		0.83	0.38		0.26	0.10		0.12	0.04				
Lentisphaerae	1				0.26	0.16		1.34	0.36	^*^	0.79	0.23			^*^	
	2				0.09	0.09		0.40	0.19		1.10	0.35				*0.09*
Verrucomicrobia	1				0.19	0.11		0.54	0.16	^**^	0.54	0.07			*0.07*	
	2				0.02	0.02		0.07	0.03		0.50	0.07				^**^
Spirochaetes	1				0.37	0.16		0.42	0.10		0.30	0.06		^*^		
	2				0.14	0.01		0.14	0.05		0.37	0.10		^**^		^*^
Planctomycetes	1				0.17	0.08	^*^	0.33	0.12	^*^	0.45	0.11		^*^		
	2				0.01	0.01		0.03	0.02		0.58	0.23				^*^
SR1	1							0.003	0.002	*0.05*	0.06	0.06				
	2				0.03	0.01		0.060	0.026		0.01	0.00		^*^		*0.06*
Cyanobacteria	1				0.05	0.02	^*^	0.06	0.04		0.52	0.18				^*^
	2				0.01	0.01		0.05	0.05		0.30	0.10				*0.05*
Elusimicrobia	1										0.34	0.23				
	2										0.60	0.57				
Chloroflexi	1				0.02	0.01	^**^	0.04	0.01		0.12	0.05		^**^		
	2							0.02	0.01		0.21	0.07				^*^
Fusobacteria	1							0.002	0.001		0.005	0.003				
	2							0.002	0.001		0.006	0.004				

a At 15 weeks of age, lambs were gavaged with fresh (group 1) or freeze-thaw (group 2) rumen microbial inocula from adult sheep. From week 21 through 26 lambs received daily a mild dose of ochratoxin A, and from week 27 lambs were in a common pen.

* P < 0.05,

** P < 0.01.

Trends (P < 0.10) are indicated with actual P-values. Comparisons were made between groups for each period and within groups at different periods (n = 4).

**Figure 1:**
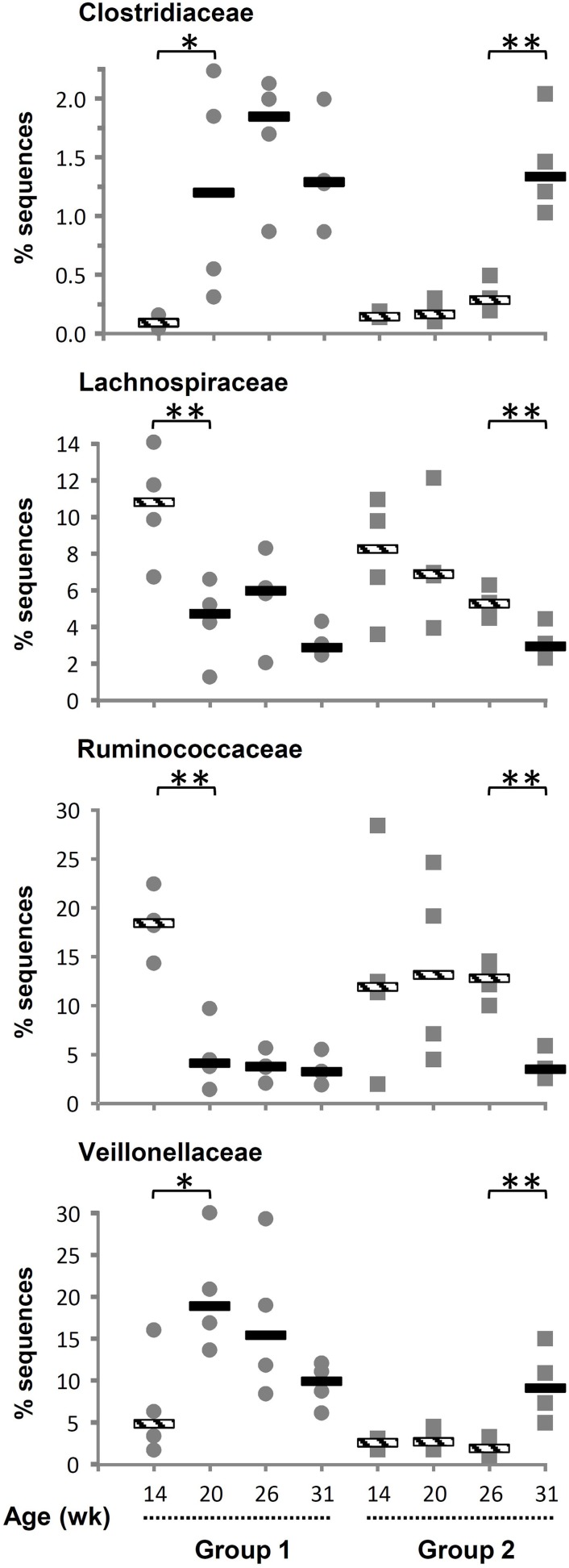
**Variation in relative abundance of bacterial families of as a function of interventions modulating the rumen microbiota in standard (1) and “stressed” microbiota (2) groups**. Values for individuals and the median are shown. Striped and solid rectangles indicate, respectively, absence or presence of protozoa. ^*^P < 0.05, ^**^P < 0.01.

The number of phyla increased following inoculation but also with age, going from only six phyla detected in 14-week-old lambs up to 14, 15, and 16 phyla at 20, 26, and 31 weeks of age, respectively (Table 2). Once a phylum was detected it remained in following sampling times suggesting that they were autochthonous to the environment. TM7 and Armatimonadetes were also detected but not further considered because they appeared in less than half of the animals at a given sampling period. When sequences were analyzed at a lower taxonomical level, i.e., families representing at least 0.1% of the sequences at a given sampling time, the majority of bacteria were classified into the Prevotellaceae from the Bacteroidetes phylum (mean 40%). The Firmicutes were more diverse and were represented by Veillonellaceae (mean 8.9%), Ruminococcaceae (mean 9.3%), and Lachnospiraceae (mean 5.9%) as the largest groups. Fibrobacteraceae were also abundant but results were similar to those presented above for the phylum as, as expected, nearly all Fibrobacteres sequences were also classified into this family. The most important changes at the family level were associated to the presence of a diverse protozoa community in the ecosystem, as substantial variations were observed between 14 and 20 weeks of age for G1 and between 26 and 31 weeks of age for G2 (Supplementary Table S1). In contrast, exposure to OTA impacted the bacterial community less, as judged by fewer changes observed in populations and β-diversity analysis (see below). In association with a normal density and diversity of protozoa, Prevotellaceae decreased by ~40% while Ruminococcaceae and Lachnospiraceae decreased 2–6 folds. In contrast, Clostridiaceae and Veillonellaceae increased 3–10 folds (Figure 1). For some of these families, the changes could be largely attributed to known genera like *Prevotella* for Prevotellaceae, *Clostridium* for Clostridiaceae or *Selenomonas* and *Succiniclasticum* for Veillonellaceae. However, for Lachnospiraceae the decrease could be only partially attributed to *Roseburia* and in the case of Ruminococcaceae it could not be attributed to any identified genus. Quantitative PCR using species-specific primers confirmed that *F. succinogenes*, undetected in 14-week-old lambs, was an important species in older lambs (mean 7.2 log_10_ copies 16 s rRNA gene/mg DNA, *P* < 0.05). In contrast, changes in Veillonellaceae could not be attributed to *S. ruminantium*, which remained constant throughout the experiment (mean 7.4 log_10_ copies 16 s rRNA gene/mg DNA, *P* > 0.05). The qPCR data also confirmed that decreases in Ruminococcaceae numbers could not be attributed to the common fibrolytic genera, *R. albus* and *R. flavefaciens* that showed no changes or even a tendency to increase for the latter. Total bacteria, 16S rRNA gene copies per unit of DNA were logically higher in the absence/low density of protozoa (Supplementary Figure S4).

**Figure 2:**
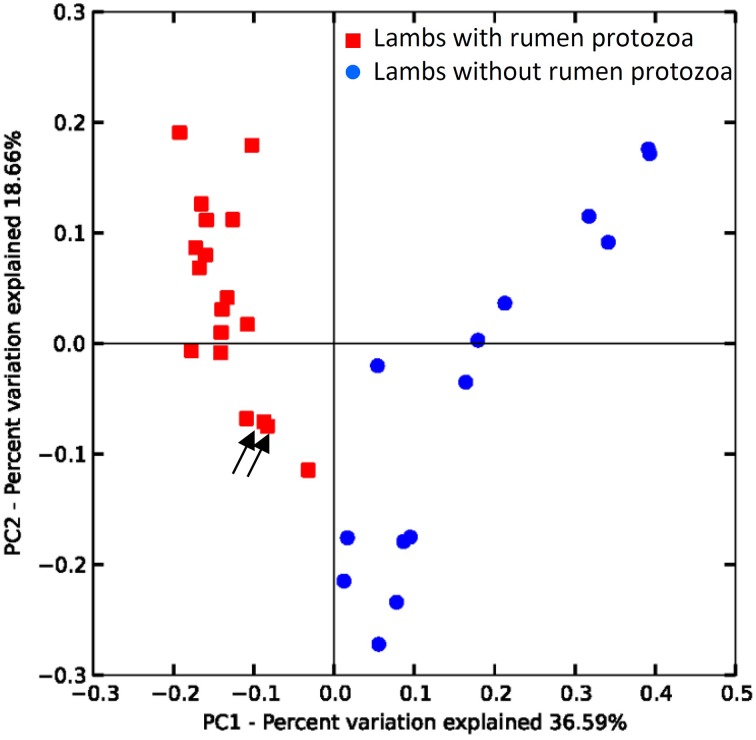
**Differences in the rumen bacterial communities of lambs due to the presence/absence of protozoa**. PCoA of weighted UniFrac distances. Arrows indicate protozoa contaminated lamb from G2 at 20 and 26 weeks (see text).

Beta-diversity analysis using the UniFrac metric (Lozupone and Knight, 2005) revealed that the presence of protozoa was the main discriminant parameter for the bacteria community, particularly influencing the relative abundance of taxa (Figure 2 weighted UniFrac) but also affecting the presence of rarer taxa (Supplementary Figure S5 unweighted Unifrac plot). The effect of challenging events like adult microbiota inoculation and OTA contamination in the diet was assessed for each group by comparing the Bray-Curtis similarity for the same animal at different time points. For G1 the lowest similarity was observed between 14 and 20 weeks of age at 56% following inoculation with fresh rumen content inoculum. This similarity value was different (*P* > 0.05) to 69 and 73% for 20 vs. 26 and 26 vs. 31 weeks, respectively. For G2 the lowest similarity, although not statistically different from the other periods, was also observed when lambs were allowed to be colonized by protozoa (26 vs. 31 weeks = 61%). The similarity between 14 and 20 and between 20 and 26 weeks of age were 63 and 72%, respectively. Protozoa seemed to produce the biggest shift in bacterial β-diversity substantiating the Unifrac results.

### Rumen archaea

**Figure 3:**
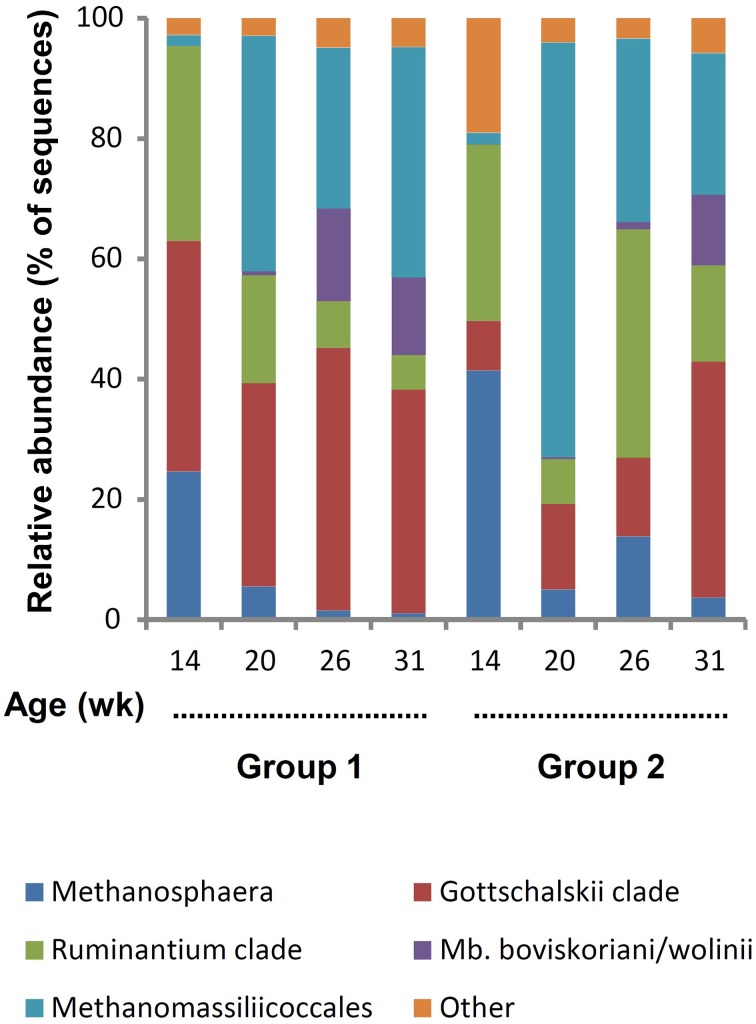
**Color coded bar plot showing average archaeal clades distribution in lambs as a function of interventions modulating the rumen microbiota in standard (1) and “stressed” microbiota (2) groups**.

For archaea, a total of 78,517 reads were retained with an average per sample of 2454 ± 1179. The minimum and maximum counts per sample were 226 and 4912, respectively. The span between samples was proportionally larger than for bacteria and, in archaea's case, differences were observed between groups (*P* = 0.0373). There was a general trend for increased counts with aging, pairwise comparison between groups revealed that the difference was due to variance between 14 and 31 weeks for G1. Both the average Good's coverage estimator of 99.6 ± 0.4% and leveled off rarefactions curves (Supplementary Figure S2B) indicate that the number of sequences generated was adequate. The Shannon diversity index increased with aging (*P* > 0.05) and G2 was less diverse than G1 for the first three sampling ages (Table 1). This difference between groups was significant at 20 weeks of age (*P* < 0.05). A total of 77 OTUs were identified at 97% similarity but many of these OTUs were present in low numbers and only in some lambs and were not further considered. Based on their abundance, 29 OTUs were retained as they represented ≥1% of the total number of archaeal sequences and were present in ≥50% of the rumen samples from a group at a given time (Supplementary Table S2). These were grouped using the main clades reported in the rumen (Janssen and Kirs, 2008; Figure 3). It was observed that *Methanosphaera* were important at 14 weeks but decreased following rumen inoculation. In contrast, Methanomassiliicoccales were a minor group at 14 weeks but became predominant in both groups following inoculation with rumen fluid from adult sheep. The *Mbb. boviskoriani/wolinii* clade increased in older animals and with a standard protozoal community.

**Figure 4:**
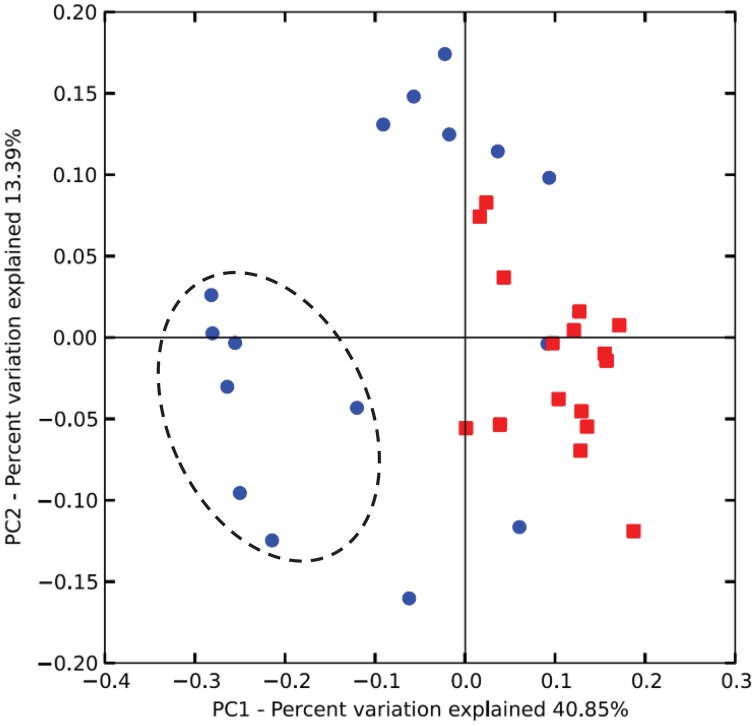
**Differences in the rumen archaeal communities of lambs as a function of interventions modulating the rumen microbiota**. PCoA of unweighted UniFrac distances. As for Figure 2, squares and circles indicate lambs with and without protozoa respectively. Dotted circle indicates lambs at 14 weeks of age.

Unweighted Unifrac analysis (performed with all 77 OTUs) discriminated on the first axis samples from lambs at 14 weeks from those taken at other periods indicating a less marked effect of protozoa on methanogens than on bacteria. The combined effect of age and inoculation were more important determinants of the archaeal community than protozoa alone (Figure 4).

### Metabolic phenotype profile

The evolution of lambs' metabolic phenotypes throughout the experiment was monitored in urine using LC-Q-ToF untargeted metabolomic analyses in combination with multivariate methods. All data, including quality control samples (QCs) that were included throughout the analysis, were first examined by PCA to obtain a global overview of the different sources of variability. A clear separation between lambs mainly explained by the presence or absence of protozoa was observed along the first component highlighting the massive effect of colonization events on urinary metabolites (Supplementary Figure S7). An arched trajectory was observed, similarly to bacterial community profiles shown in Figure 2.

**Figure 5:**
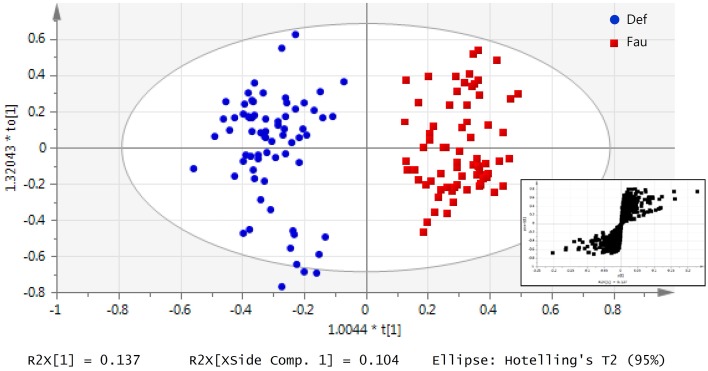
**Urinary metabolic profile of lambs as a function of interventions modulating the rumen microbiota in standard (G1) and “stressed” microbiota (G2) groups represented by an OPLS-DA model of all lambs throughout the 31 weeks experiment**. Data was classified by the presence or absence of protozoa. At each sampling period at 14, 20, 26, and 31 on weeks of age, urine was daily collected for up to 5 days. Each data point represents one lamb/day, for a given period each lamb was analyzed between 3 and 5 times. Inset: S-plot from OPLS-DA model highlights relevant metabolites according to the amplitude p[1] and reliability p(corr)[1] of their variations.

**Figure 6:**
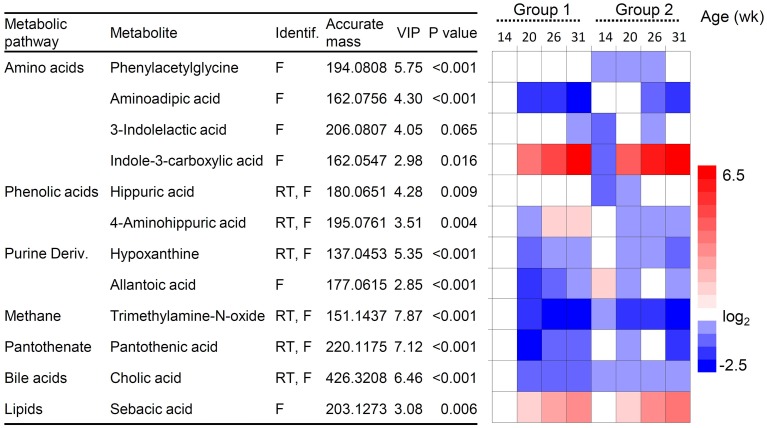
**Changes in urine metabolites in lambs of group 1 and 2**. Metabolites were selected from the OPLS-DA analysis (variable importance of the projection, VIP-values). *P*-values indicate differences between presence/absence of protozoa analyzed by ANOVA test. Heatmap shows changes in metabolites relative to group 1 at 14 weeks of age taken as the baseline. Red and blue hues represent fold increase and fold decrease, respectively. Metabolites were identified by their molecular mass, retention time (RT) and MS/MS fragmentation spectrum (F).

Supervised multivariate analysis was then carried out using OPLS-DA to focus on discriminative metabolic patterns between faunated and non-faunated animals. The assessment of a global OPLS-DA model accounting for all time points resulted in one predictive and two orthogonal components (Figure 5), with a total explained variance R^2^X(cum) of 32.8% and a cross-validated predictive ability Q^2^Y(cum) of 78.1%. The explained variance was split into predictive variation (R^2^p(X) = 13.7%) and the uncorrelated variations (orthogonal variation, R^2^to(X) = 19.4%). A separate comparison of samples at each time point was then made using PCA and OPLS-DA models. They showed negligible differences between the two groups of lambs at 14 weeks of age despite the fact that they were physically separated but, otherwise, had the same initial contact with their dams and were similarly treated. At 20 and 26 weeks of age both groups were well separated, similarly to the global PCA and OPLS-DA models (figures not shown). However, at 31 weeks, when lambs were all in the same lot, previous differences were effaced. The most discriminant metabolites were selected using a VIP threshold of 2. Figure 6 shows identified metabolites and their time course trajectory calculated using the relative concentrations and their changes in abundance compared to G1 at 14 weeks. Most abundant changes were related to metabolic pathways involving protein, amino acid and polyphenol metabolism. It should be noted that many discriminant metabolites could not be identified using well-curated databases such as METLIN and HMDB. These are shown in Supplementary Figure S8.

**Figure 7:**
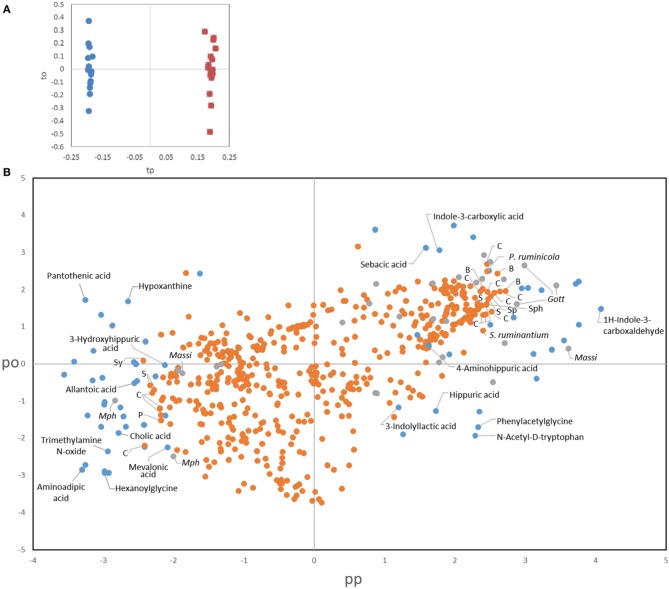
**Consensus OPLS-DA results**. **(A)** Score plot of the observations on the predictive (tp) and the orthogonal component (to). Defaunated animals are symbolized by blue dots and faunated animals by orange dots. **(B)** Combined loading plot of the three sources of data. Dots represent metabolites (blue), bacterial OTUs (orange), and archaeal OTUs (gray). Identified metabolites are labeled in the figure. Labels for the most discriminant microbial OTUs indicate the closest relative obtained by BLAST. C, Clostridiales; B, Bacteroidales; S, Selenomodales; Sp, Spirochaetales; Sy, Synergistales; Sph, Sphingobacteriales; P, Gammaproteobacteria; Massi, Methanomassiliicoccales; Mph, *Methanosphaera*.

To better comprehend the changes induced by treatments we also applied a novel data fusion approach for the joint analysis of metabolomic and microbial data, namely consensus OPLS-DA analysis (Boccard and Rutledge, 2013). Consensus OPLS-DA was performed on the most relevant information from the three sources of data, i.e., urinary metabolites, bacteria and archaea, for assessing the effect of protozoa through the comparison of all the fauna-free and faunated animals. The metabolite dataset was reduced to include the most significant metabolites (VIP > 2, *n* = 65). A stringent trimming was performed on the bacterial OTUs for obtaining a core set of phylotypes. Only OTUs that were in ≥50% of the rumen samples from a group at a given time and that represented ≥0.1% of the total community counts were selected. From an initial 3946 OTUs, 517 fitted these criteria. For archaea, the dataset contained the 29 OTUs described in the text. A model with two latent variables (one predictive and one orthogonal component) was obtained using leave-one-animal-out cross validation with satisfactory goodness of fit (R2Y = 0.95) and goodness of prediction indices (Q2 = 0.78). The faunation effect was clearly visible on the score plot of the two predictive latent variables (tp1 vs. tp2), separating the two groups (Figure 7A, fauna-free on the left and faunated on the right). The loadings allowed links between urinary metabolites and rumen microbial data (bacteria and archaea) to be drawn. This analysis confirmed the results obtained using different statistical tools for the individual datasets presented above but also provides a more integrative overview of potential links between rumen microbes and the host urinary metabolome (Figure 7B). For instance, extreme loading values for metabolites associated to tryptophan pathway where not only linked to the presence of protozoa but also to bacterial OTUs distantly related to Clostridiales and Bacteroidales. To note also that for the 25 most extreme bacterial OTUs, only two could be assigned to known species (*Prevotella ruminicola*, 98% identity, and *Selenomonas ruminatium*, 100% identity) and for the rest the closest relatives had between 95 and 82% identity. These observations are further described in the Discussion Section.

### Colonization events induced profound differences in rumen fermentation traits but without influencing growth

Table 3 shows the changes in rumen fermentation, intake, digestibility and body weight throughout the experimental period. Total VFA were greatly influenced by inoculation with rumen fluid from adult sheep. Concentration of rumen VFA increased two folds in G1 at 20 weeks compared to 14 weeks while in G2 the increase was less pronounced. Fauna-free lambs had higher proportion of propionate, lower proportion of butyrate and lower ammonia concentration. In contrast, total dry matter digestibility was negatively affected by protozoa (*P* = 0.0012), particularly in week 26 in conjunction with OTA challenge with a 6% difference observed between G1 and G2, with and without a standard protozoal population, respectively. Despite the differences described above, growth was not different between groups.

**Table 3:** **Evolution of weight, feed intake and digestibility and rumen fermentation characteristics in two groups of lambs with differing rumen microbial intervention histories^a^**.

**Measure**	**Group 1**				**Group 2**					***P*****-value**			
	**Fauna-free 14 week**	**Faunated 20 week**	**Faunated 26 week**	**Faunated 31 week**	**Fauna-free 14 week**	**Fauna-free 20 week**	**Fauna-free 26 week**	**Faunated 31 week**	**SEM**	**Age**	**Group**	**AxG**	**Protozoa**
Weight (kg)	27.4	33.8	43.4	50.1	26.0	34.9	44.7	49.8	2.20	< 0.001	0.96	0.096	0.71
Intake (g DM/d)	1263	1492	1495	1512	1329	1538	1670	1728	90.8	< 0.001	0.25	0.53	0.34
Digestibility (%)	72.1	68.0	62.5	72.6	73.5	70.4	68.8	73.8	1.17	< 0.001	0.011	0.15	0.001
Total VFA (mM)	26.3	59.0	67.9	58.7	21.3	36.4	37.4	55.4	3.69	< 0.001	0.005	0.001	< 0.001
Acetate (Ac.; %)	68.1	63.0	64.4	65.4	66.3	63.2	65.6	65.7	1.52	0.04	0.98	0.69	0.69
Propionate (Pr.; %)	20.0	19.5	19.1	19.5	20.4	24.3	23.9	19.3	1.22	0.13	0.056	0.060	0.003
Butyrate (%)	6.2	12.2	11.9	10.3	5.7	6.5	5.6	10.4	0.91	< 0.001	0.028	< 0.001	0.001
Iso VFA	3.7	4.0	3.6	3.6	5.4	4.3	3.7	3.6	0.53	0.21	0.28	0.30	0.76
Valerate + caproate	2.0	1.3	1.1	1.1	2.3	1.7	1.2	1.1	0.20	< 0.001	0.30	0.65	0.25
Ac./Pr. Ratio	3.5	3.4	3.4	3.4	3.4	2.6	2.8	3.5	0.27	0.27	0.14	0.29	0.021
Ammonia mg/L	4.8	10.1	8.7	7.8	6.8	4.2	4.6	8.1	0.74	0.047	0.018	< 0.001	< 0.001

a At 15 weeks of age, lambs were gavaged with fresh (group 1) or freeze-thaw (group 2) rumen microbial inocula from adult sheep. From week 21 through 26 lambs received daily a mild dose of ochatoxin A, and from week 27 lambs were in a common pen. The presence and abundance of protozoa influenced the results and the terms fauna-free and faunated are used to simplify the presentation.

## Discussion

The composition of the rumen microbiota of the isolated lamb groups at the first measurement was markedly different of the expected microbiota of lambs reared in contact with adult ruminants. The low diversity of bacterial phyla and the absence of protozoa indicate that the experimental conditions succeeded in maintaining a microbiota that resembled that of 2 to 4-week-old ruminants (Fonty et al., 1987, 1989; Li et al., 2012; Rey et al., 2014). The high proportion of Proteobacteria, compared to older animals, and large individual differences in the abundance of some populations observed in 14-week old lambs was also previously reported in young ruminants (Fonty et al., 1987; Li et al., 2012; Jami et al., 2013). In week 15, lambs in both groups were colonized by gavage that allowed to use the same donor sheep and amount of inoculum for all animals. The microbial composition of the inoculum could not be characterized. However, measuring changes in the inoculum and the stabilized microbiota after gavage was not an objective of the work and based on the results it can be concluded that the microbiota after gavage corresponds to the expected composition for ruminants (Jami et al., 2013; Kittelmann et al., 2013).

Young lambs at 14 weeks of age had a lower bacterial and archaeal α-diversity and a greater inter-individual variation in β-diversity (looser grouping in PCoA plots) than at later time points. It is possible that differences observed in bacterial communities between G1 and G2 at weeks 20 and 26 could be influenced by the freeze-thaw treatment imposed to the inoculum used in G2. Freeze-thaw might have affected some populations but only marginally as suggested by the absence of α-diversity differences between G1 and G2. β-diversity discrimination between groups was mainly driven by the relative abundance of taxa rather than their presence/absence indicating that freeze-thaw was not deleterious to major bacteria. It is interesting to note the contrasting abundance at different measuring periods between Fibrobacteres and Ruminococcaceae, two bacterial groups associated to plant fiber digestion. Fibrobacteres, absent in 14 week-old lambs, were not acquired during the first few hours after birth, when in contact with the mother, or through the environment. Their absence at 14 weeks and presence in older lambs was confirmed by qPCR targeting *F. succinogenes*. The qPCR results at 20–31 weeks are in line with expected values (Mosoni et al., 2011) but Ion Torrent PGM sequencing indicated a much higher abundance. The great proportion of Fibrobacteres might be due to a sequencing platform effect that might affect some taxa more than others (Salipante et al., 2014) but this need to be confirmed. The *Ruminococcaceae* were highly represented in 14-week-old lambs but the majority of sequences were not attributed to common fibrolytic spp. *R. albus* and *flavefaciens*.

Except for *Methanomicrobium* spp, all main methanogens clades described in the rumen (Janssen and Kirs, 2008) were present throughout the entire experimental period. However, for some lambs the proportion varied widely. The most striking change observed in the methanogens' community was the negative relationship between the genus *Methanosphaera*, abundant at 14 weeks, and the Methanomassiliicoccales that became abundant at later sampling periods in both groups. Additional species were introduced when lambs were inoculated with sheep microbiota diversifying the Methanomassiliicoccales population but the apparent blooming of some species between 14 and 20 weeks could also be explained by changes in the microbial, mainly bacterial community, resulting in changes in substrates required for methanogenesis. Differently from most rumen methanogens, these two methanogens cannot utilize CO_2_ and H_2_. *Methanosphaera* uses only methanol and H_2_ as methanogenic substrates (Fricke et al., 2006) and members of the Methanomassiliicoccales can also use tri-, di-, and mono-methylamine compounds (Borrel et al., 2012). Recently, the presence of gene transcripts involved in methanol and methylamines utilization were identified in the rumen and associated to the activity of Methanomassiliicoccales (Poulsen et al., 2013). *Methanosphaera* and the new order Methanomassiliicoccales occupy similar trophic niches but the more versatile use of substrates by the latter may explain their higher abundance. Opposed to these results, Kittelmann et al. (2013) reported a positive correlation between these populations in ruminants from New Zealand. After the introduction of a standard protozoal community, some OTUs assigned to *Mbb. ruminantium* decreased while others from the *Mbb. gottschalkii* clade and Methanomassiliicoccales increased. However, we did not find clear evidence of a distinct community of methanogens when protozoa were present or absent as reported by other authors (Ohene-Adjei et al., 2008). Differences may be due to animal experimental conditions or use of primers (Tymensen and McAllister, 2012). Tymensen et al. (2012) reported that for cattle *Methanobrevibacter* spp. were more commonly associated to protozoal cells while Methanomassiliicoccales were more abundant in the liquid fraction.

### Modification of host metabolic phenotype by rumen microbes and colonization history

Throughout the study, there was an association between the structure and composition of the rumen microbiota and urine metabolome. In particular, bacteria and protozoa were the populations that had the greatest effects. Additional rumen biochemical characteristics and other measures on the host animal were also affected by the microbiota. Interesting insights can be inferred when considering the rumen microbial status and phenotypes with practical economic value such as weight gain and digestibility. The immature microbiota at 14 weeks, devoid of some keystone rumen members such as the fibrolytic *F. succinogenes*, did not have a negative effect on lambs' growth as compared to lambs of the same genetic stock reared under normal conditions in the same experimental farm (M. Bernard, personal communication). Total tract digestibility was also adequate for this type of diet, although it is noted that the measure does not differentiate between rumen and the intestine and less rumen activity may be compensated by greater digestion in the hindgut. Rumen fermentation was stimulated by the microbial inoculum, particularly for G1 containing protozoa. This is in accord with the microbial ecology tenet that higher diversity would improve ecosystems functions and stability (Konopka, 2009). However, protozoa negatively affected digestibility, particularly during the OTA challenge. This might be independent of the supposedly high OTA-detoxifying activity of protozoa (Mobashar et al., 2010) but it rather suggests a lower post-ruminal digestibility that remains to be confirmed.

In addition to the effect on rumen fermentation parameters, microbial inoculation also profoundly modified the host urinary metabolome. Most discriminant metabolites were produced by microorganisms or were microbial metabolites modified by the host (co-metabolites). Some clearly increased following inoculation with the complex rumen microbiota such as sebacic acid or indole-3-carboxylic acid, a metabolite associated to tryptophan metabolism, and while others decreased, such as trimethylamine N-oxide (TMAO). Trimethylamine N-oxide is synthesized in the liver from trimethylamine produced by gastrointestinal microorganisms from dietary sources. In ruminants, trimethylamine is a common rumen metabolite that is mainly originated from dietary choline and glycine-betaine (Neill et al., 1978; Mitchell et al., 1979). In humans, choline, betaine and TMAO are implicated in atherosclerosis (Wang et al., 2011) with methanogens of the order Methanomassiliicoccales hypothetically having a protective role in this pathology (Gaci et al., 2014). Our animal model shows indeed that there is a negative relationship between gut Methanomassiliicoccales and urine TMAO. In ruminants the interest is vested in the role of this group of methanogens in methane production; the results hints that TMAO could be a potential biomarker of methylamine-utilizing rumen methanogens.

The presence of protozoa also seems to modify the host metabolome, either because of their own metabolic activity or by modulating the bacterial community. Purine derivatives, allantoic acid and hypoxanthine, are markers of microbial protein synthesis (Chen and Gomes, 1992) and their decrease in the presence of protozoa is attributed to lower bacterial biomass due to predation (Hristov and Jouany, 2005). Other metabolites discriminated for the presence/absence of protozoa such as aminoadipic acid, an intermediate of lysine metabolism, cholic acid and pantothenic acid. However, many other metabolites still need to be identified because there are not available standards and/or their characteristics (molecular mass, retention time and MS/MS spectrum) do not match HMDB or our in-house database highlighting the distinctiveness of the ruminant holobiont metabolism.

### Individual differences and changes in urine metabolome

Many animal species including humans exhibit individual differences in their microbiota. These differences have been ascribed to diet, intrinsic host genetic and immunological factors and to ecological processes of microbial community composition (Mulder et al., 2011; Costello et al., 2012). Controlled animal experiments, as the one described here, can highlight the importance of some of these processes. Inoculation with a “stressed” microbiota in G2 resulted in only one lamb harboring an incomplete protozoal community 6 weeks after the inoculation (a second lamb presented low protozoal concentration 12 weeks after inoculation). The successful implantation of protozoa in this lamb and, on the same account, the lack of implantation on other G2 lambs could be due to host factors but it could also be explained by environmental selection by the rumen ecosystem. It is also noted that the lamb with a different protozoal status also consistently showed different bacterial and urine metabolome profiles compared to other G2 lambs corroborating the strong interactions between microbial groups inhabiting the rumen and the host. In addition, the quick acquisition of protozoa for the rest of G2 lambs when in contact with G1 suggests that exposure to a diverse protozoal community facilitated dispersal, probably through the creation of new ecological niches (Costello et al., 2012). Differences in the microbiota and metabolic urinary profiles between groups were effaced 6 weeks after all lambs were mingled in a same pen. In contrast to other reports (Yáñez-Ruiz et al., 2010), the absence of enduring differences between groups observed in this experiment could be because colonization events occurred several weeks after birth, well after the rapid immunological and anatomical development of the digestive tract GIT. In addition, there were not reinforced by dietary changes or diet differences.

We observed that growth in lambs was not affected by a reduced rumen microbial diversity, by the presence or not of protozoa or by a mild stress treatment. In spite of that, rumen microbial diversity had a major influence on rumen fermentation parameters, digestibility, and the general metabolism of the host as monitored through the urinary metabolome profile. These results suggest that under controlled and unchanged sanitary, environmental, and dietary conditions an adequate rumen function can be provided by a microbiota with reduced diversity. The evolutionary advantages for ruminants to maintain an extremely diverse microbial community in the rumen could be regarded as an insurance policy against dietary changes and occasional aggressions such as xenobiotics. The interaction found between TMAO and methanogens from the order Methanomassiliicoccales shows potential for the metabolomics approach for the discovery and monitoring of biomarkers of rumen microbial functions of interest.

### Conflict of interest statement

The authors declare that the research was conducted in the absence of any commercial or financial relationships that could be construed as a potential conflict of interest.
