# Supplementary material for: Rumen microbial communities influence metabolic phenotypes in lambs
Source: Front Microbiol. 2015 Oct 12;6:1060. doi: 10.3389/fmicb.2015.01060 (PMC4601264; doi:10.3389/fmicb.2015.01060)
Supplement: Supplementary file 1 [file SupplementaryMaterial.PDF]

Supplementary Material

Rumen microbial communities influence metabolic phenotypes in lambs

Diego P. Morgavi*, Estelle Rathahao-Paris, Milka Popova, Julien Boccard, Kristian F. Nielsen, Hamid Boudra

*** Correspondence:** diego.morgavi@clermont.inra.fr

1. Supplementary Materials and Methods

1.1. Digestibility and sampling procedures

Individual dry matter intake (DMI) and total tract digestibility were measured during 4 days at the end of each period. Lambs were adapted to metabolic cages before each measurement. Feed intake and orts were recorded daily during the sample collection period to calculate dry matter intake (DMI). The cages allowed the separation of urine and feces. Total tract digestibility was determined from the total collection of feces during the measurement period. Every day fecal samples were weighed, mixed and a 10% aliquot was sampled. Daily feed and fecal samples from the 4 days were pooled by animal; one aliquot was used for dry matter determination (103 °C for 24 h)

During the digestibility period, urine was daily collected in clean containers containing sodium azide (0.5 g/L urine). A 10 mL sample was then immediately centrifuged (4000 × g, 10 min), 1-ml supernatant aliquots were dispensed in 2 ml polypropylene tubes, and stored at –80°C until analysis.

Rumen content samples for fermentation and microbiological parameters were taken twice for each period. Sampling was done using an esophageal tube before the morning feeding on the days before and after the digestibility measures. Samples were strained through a polyester monofilament fabric (250 µm mesh aperture) to remove solids. For volatile fatty acids (VFA), 0.8 mL rumen liquid were transferred to a microcentrifuge tube containing 0.5 mL of a 0.5 N HCl solution containing 2% (w/v) metaphosphoric acid and 0.4% (w/v) crotonic acid, kept at 4 °C for 2 h, centrifuged (16,500 × g, 10 min, 4 ºC) and supernatants stored at -20 °C until analysis. For ammonia (NH_3) determination, 2 ml rumen fluid were mixed with 0.2 ml 5% (v/v) metaphosphoric acid and stored at -20°C until analysis. For protozoa, strained rumen fluid samples were mixed with methylgreen-formalin solution in a 1:1 ratio and stored at room temperature in the dark until used for protozoal counting. For microbial analysis by molecular biology, 1 ml rumen liquid filtrate was transferred to 1.5 ml microtubes, centrifuged (15000 × *g*, 15 min, 4 °C), 0.8 ml supernatant was discarded and the pellet was stored at –80° C until processing.

1.2. Analytical methods

Volatile fatty acids were analysed by gas chromatography (CP 9002 Chrompack, Middelburg, Germany) using a wall-coated open-tubular fused silica column (0.25 mm id × 25 m) coated with CP-wax 58 (FFAP)-CB (Morgavi et al., 2003). Ammonia content of rumen liquid was determined by colorimetry using the phenol-hypochlorite method (Weatherburn, 1967). The reaction was carried out in duplicate in 96-well plates using an Infinity M200 spectrophotometer (Tecan Austria GmbH, Grödig, Austria).
Quantitative PCR assays were run in triplicate using the SYBR Premix Ex Taq kit (Lonza, Levallois-Perret, France) on a StepOnePlus system (Applied Biosystems, Foster City, CA). Negative controls without DNA template were run with every assay to assess the overall specificity. The PCR amplification efficiency was checked as described before (Mosoni et al., 2011) using standard curves, 10^8 to 10^3 copies prepared from 16S rRNA gene fragments amplified from genomic DNA of *Prevotella bryantii*. This bacterium was used as standard as the amplification curve did not differ from the curve produced by a mixture of eleven representative rumen bacterial species (Mosoni et al., 2011). The slope and efficiency for 16S rRNA primers for total bacteria, *Fibrobacter succinogenes*, *Ruminococcus albus*, *R. flavefaciens*, and *Selenomonas ruminantium* were: -3.446 and 95.1%, -3.190 and 105.8%, -3.544 and 91.5%, -3.520 and 92.3%, 3.312 and 100.4%, respectively. PCR efficiency with DNA samples was confirmed to be similar to that obtained with the standard curves. For each rumen content sample, results were expressed as the mean of 3 replicates in 16S rRNA gene copies per mg of DNA.

1.3. Liquid chromatography-mass spectrometry (LC-MS) analysis of urine

Urine and QC samples were thawed at room temperature. A 200-µl aliquot was transferred into a clean polypropylene tube and mixed with 200 µl of 2% formic acid. The samples were vortex-mixed and centrifuged at 10 000 g for 10 min, then the supernatants were transferred into an autosampler vial, and an aliquot of 10 µl was injected into a LC–MS system (Nielsen et al., 2011). The separation was performed on a 100 × 2 mm i.d. Luna column (Phenomenex, Torrance, CA) fitted with a same type 2 × 2 mm guard column. The analysis was done at a flow rate of 0.3 mL/min at 40 ˚C with a water/acetonitrile (both containing 20 mM formic acid) gradient, starting at 0% going to 100% of acetonitrile in 20 min, maintaining at 100% acetonitrile for 5 min prior to returning to the starting conditions in 3 min with equilibration for 5 additional min before the next injection. The MS system was operated in the positive ESI mode and tuned to a resolution >6000 (full width at half peak height) on the reserpine solution at a flow of 50 µl/min infused with a Havard Apparatus 11 syringe pump (Holliston, MA, USA). The capillary was held at 3000 V and the cone 1 (skimmer 1) and the cone 2 (skimmer 2) at 30 V and at 6 V, respectively. The desolvation temperature was maintained at 350 °C during the run at 0.3 ml/min and the source was kept at 120 °C. The LockSpray was operated with a cone voltage of 35 V spraying a solution of 0.2 µg/ml of leucine enkephalin in acetonitrile–water–formic acid (50:50:0.1) at a flow of 4 µl/min from the syringe pump. The m/z 556.2771 $[M+H]^+$ ion from leucine enkephalin was used as lockmass.

Mass spectra were collected as centroid data with a scan time of 1 s and an inter scan time of 0.1 s. The performance of analytical instrumentation, especially the stability of the chromatographic system, was evaluated throughout data acquisition by running quality control samples (QCs) at the beginning, middle and the end of every run. Four types of QCs were used throughout this study. They were prepared by mixing equal volume of urine samples from one animal (#2) over the four periods.

1.4. Metabolite identification

Compound identification was carried out by manual annotation of ions related to discriminating ions (VIP>2). Redundant mass features corresponding to different types of ions (e.g., fragments and adducts) were used to help confirm the protonated species. In order to obtain the accurate masses of ions of interest, pooled urine samples were analyzed using a hybrid LTQ Orbitrap Fourier Transform mass spectrometer (LTQ Orbitrap XL Thermo Fisher Scientific, Bremen, Germany), equipped with an electrospray ionization source operated in the positive ion mode. The following conditions were used: needle voltage, 3.8 kV; heated capillary temperature, 275°C; capillary voltage, 20 V; tube lens offset, 70 V. Sheath gas and auxiliary gas were nitrogen at flow rates of 45 arbitrary units (a.u.) and 10 a. u., respectively. Mass resolving power (full width at the half maximum height, FWHM) was kept at 100, 000 for signal at m/z 400. Tandem mass spectrometry (MS/MS) experiments were performed for the ions of potential markers to get structural information. Helium was used as collision gas. The parameters used to perform CID (collision-induced dissociation) in the linear trap were as follows: isolation width of precursor ions of 1.5 u, activation time of 30 ms, and normalized collision energy of about 20 (arbitrary units). During the MS/MS experiments, precursor ion was selected and fragmented in the linear ion trap.

Three online metabolomics databases were queried using accurate mass values (± 5 ppm) to generate structural hypotheses: the Human Metabolome Database (HMDB; www.hmdb.ca), KEGG (http://www.genome.jp/kegg/) and Metlin (http://metlin.scripps.edu/). Finally, the identity of each marker was confirmed by comparing its product ion spectrum and its retention time with those of its commercially available compound (data not shown).

1.5. Preparation of ochratoxin-contaminated feed

Ochratoxin A (OTA) is a possible human carcinogen and was manipulated following appropriate safety precautions. The contaminated feed was prepared under controlled laboratory conditions using an *Aspergillus ochraceus* strain high producer of OTA. The strain was grown on wheat incubated at 28 °C for 4 weeks. At the end of the incubation period the cultures were dried at 45 °C for 48 h in a forced air oven, pooled, and ground to pass a 1 mm screen. Ochratoxins in the homogenized sample were analyzed in quadruplicate by HPLC-FLD as already described (Boudra and Morgavi, 2006). OTA concentration was 237.3 ± 16.0 µg/g (mean ± SD) and was used to calculate the dose given to sheep. There was also a weak concentration of OTB at 13.7 ± 0.5 µg/g. All feed components tested did not contain ochratoxins or other mycotoxins commonly produced by *A. ochraceus* like citrinin and penicillic acid. Fresh water and a mineral salt block were available all the time to animals.
The contaminated wheat was dispensed in gelatin capsules that were administered orally before the morning feeding using a bolus applicator. This procedure ensured the complete ingestion of the desired toxin amount.

References

Boudra, H., and Morgavi, D.P. (2006). Development and validation of a HPLC method for the quantitation of ochratoxins in plasma and raw milk. *Journal of chromatography. B, Analytical technologies in the biomedical and life sciences* 843, 295-301. doi: 10.1016/j.jchromb.2006.06.018.

Morgavi, D.P., Boudra, H., Jouany, J.P., and Graviou, D. (2003). Prevention of patulin toxicity on rumen microbial fermentation by SH-containing reducing agents. *Journal of Agricultural and Food Chemistry* 51, 6906-6910. doi: 10.1021/jf034505v.

Mosoni, P., Martin, C., Forano, E., and Morgavi, D.P. (2011). Long-term defaunation increases the abundance of cellulolytic ruminococci and methanogens but does not affect the bacterial and methanogen diversity in the rumen of sheep. *Journal of Animal Science* 89, 783-791. doi: 10.2527/jas.2010-2947.

Nielsen, K.F., Mansson, M., Rank, C., Frisvad, J.C., and Larsen, T.O. (2011). Dereplication of microbial natural products by LC-DAD-TOFMS. *Journal of Natural Products* 74, 2338-2348. doi: 10.1021/np200254t.

Weatherburn, M.W. (1967). Phenol-hypochlorite reaction for determination of ammonia. *Analytical Chemistry* 39, 971-974.

2. Supplementary Figures and Tables

2.1 Supplementary Figures

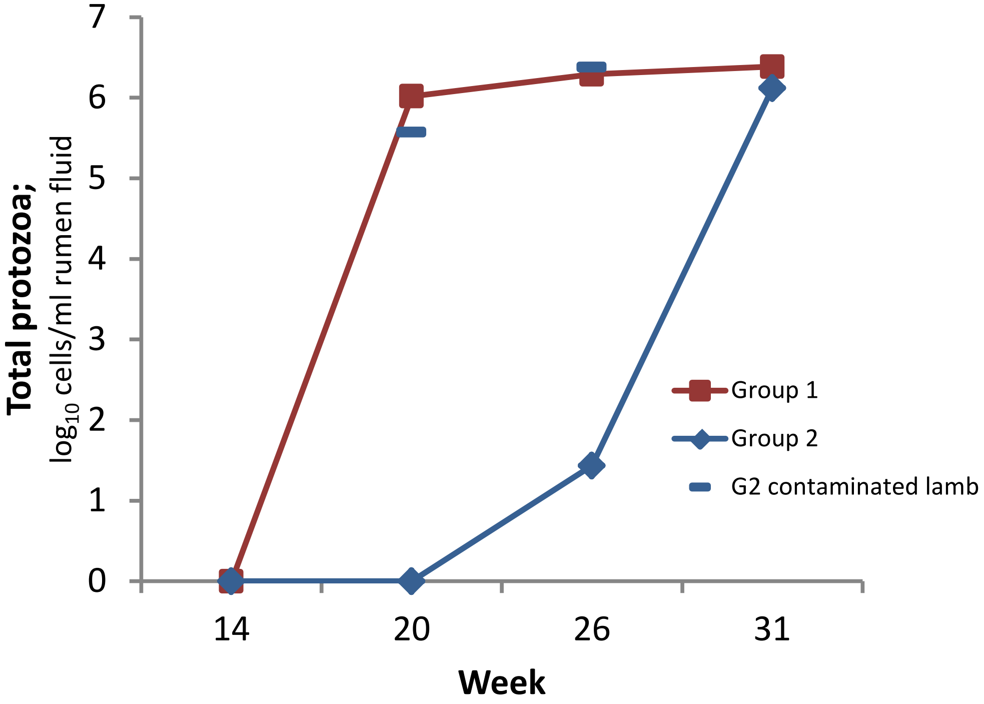

Supplementary figure S1. Total rumen protozoa concentration in lambs of group 1 and group 2. Values show the median (n=4), the blue dash indicates protozoa concentration in the only lamb of group 2 harboring protozoa at 20 weeks.

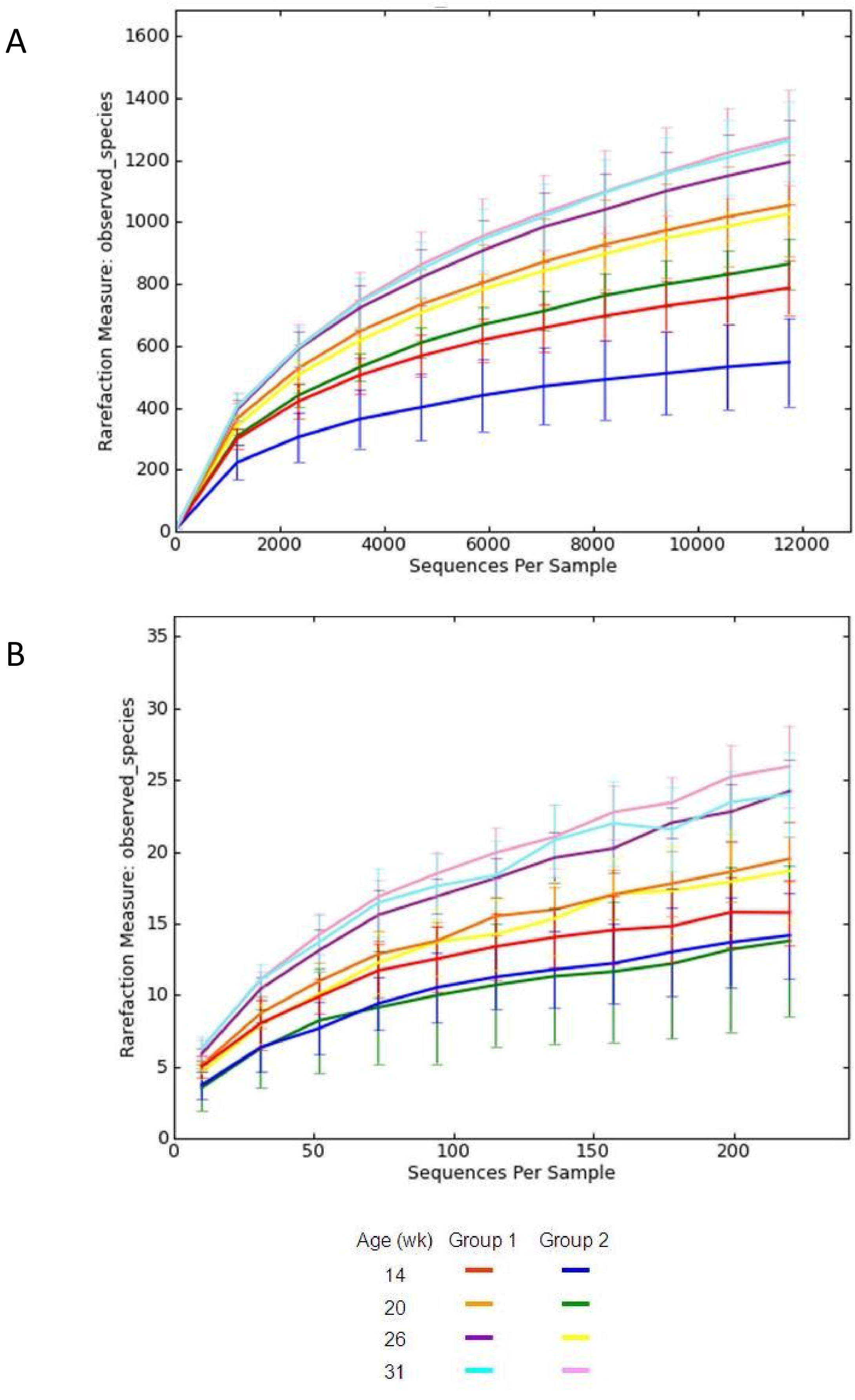

Supplementary figure S2. Rarefaction curves for bacteria (A) and archaea (B)

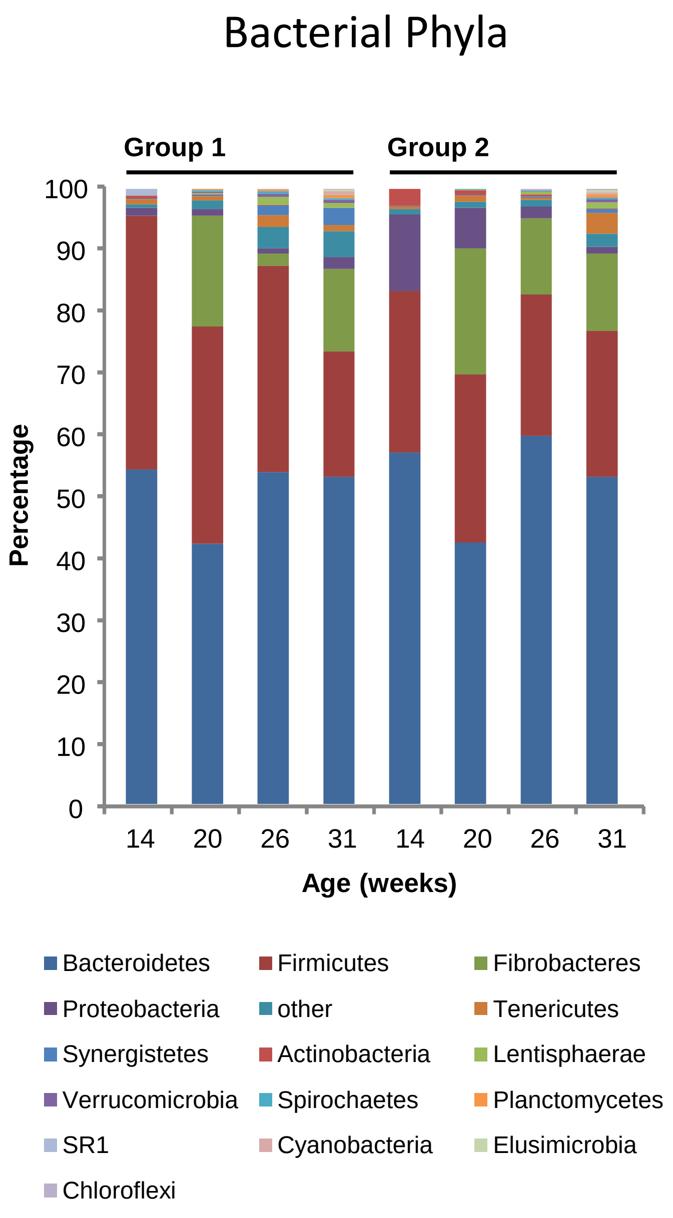

Supplementary figure S3. Relative proportion of bacterial phyla

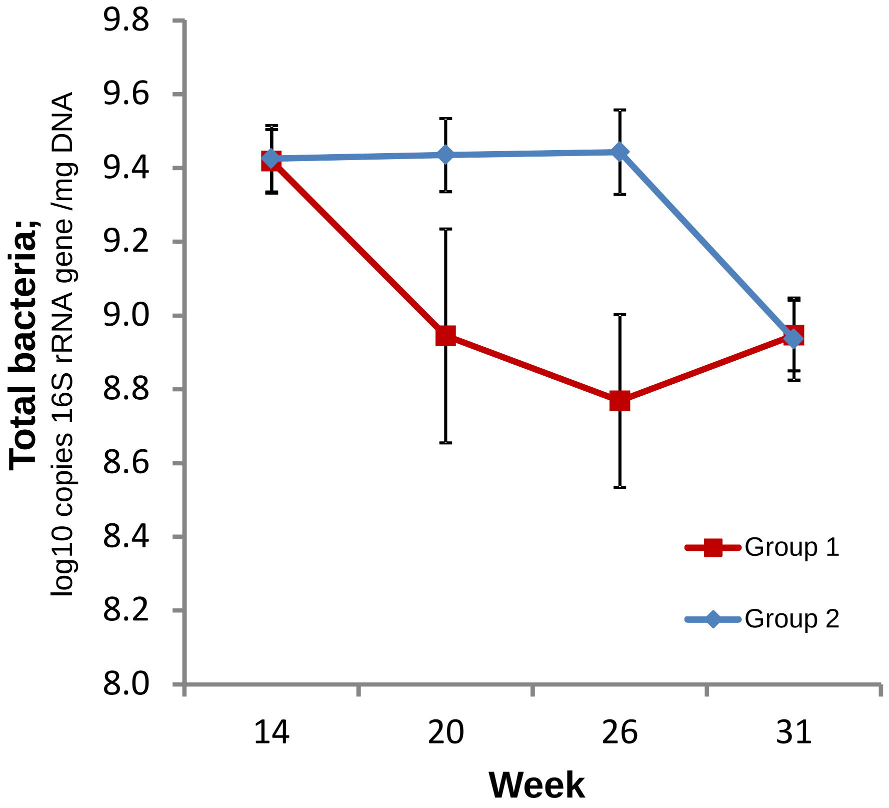

Supplementary figure S4. Amount of 16S rRNA gene copies of bacteria in lambs of group 1 and 2 throughout the experimental period. Values are means ± SD (n=4) of qPCR amplifications using universal bacterial primers.

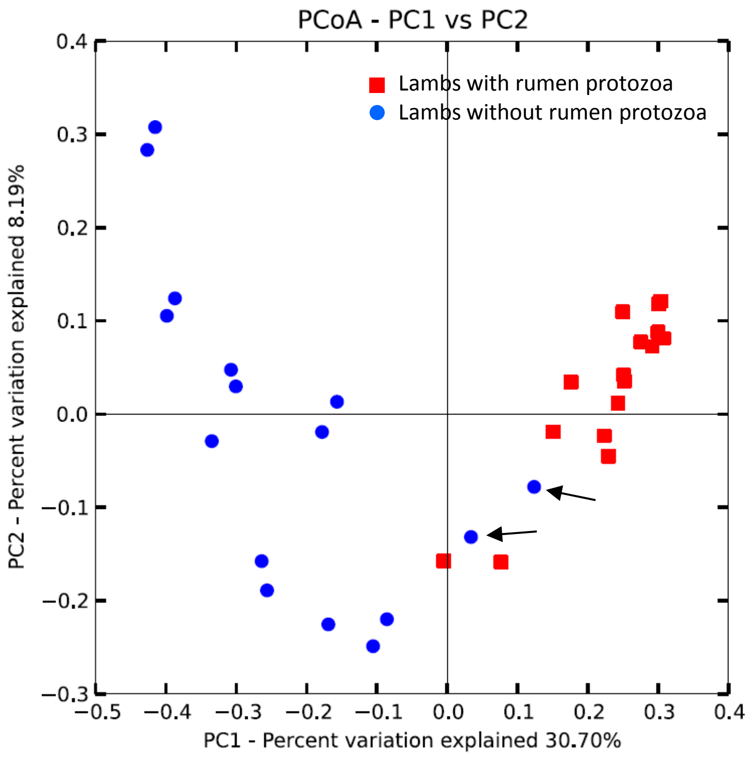

Supplementary figure S5. Differences in the rumen bacterial communities of lambs due to the presence/absence of protozoa. PCoA of unweighted UniFrac distances. Arrows indicate lamb from G2 at 20 and 26 weeks (see text).

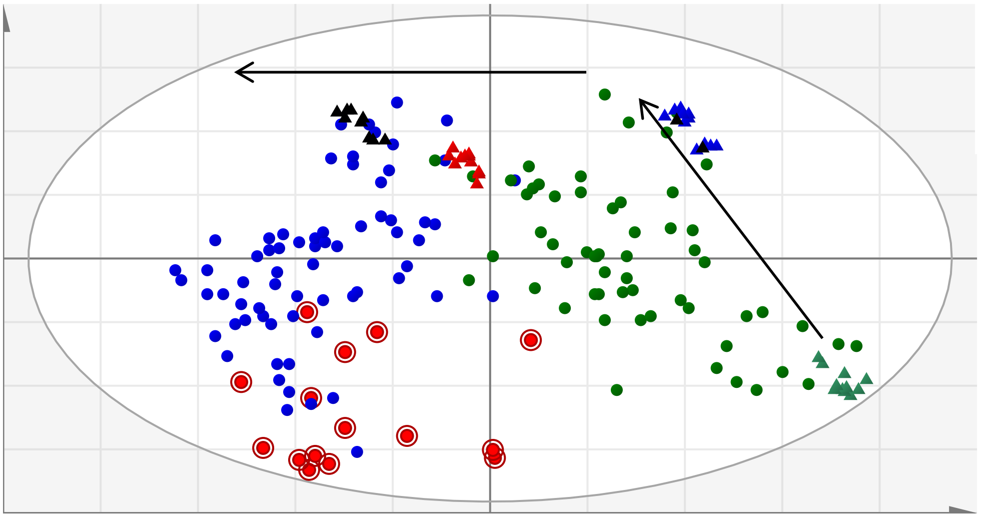

Supplementary figure S6. Urinary metabolic profile of lambs as a function of of interventions modulating the rumen microbiota in standard and ‘stressed’ microbiota groups using a PCA model. Green and blue circles represent periods without and with protozoa, respectively. Red circles show samples from protozoa-harboring lamb of G2 discussed in the text. Arrows show the trajectory of samples through time. At each sampling period at 14, 20, 26, and 31 on weeks of age, urine was daily collected for up to 5 days. Each data point represents one lamb/day, for a given period each lamb was analyzed between 3 and 5 times. Triangles are quality control (QC) samples corresponding to each sampling period. The tight clustering of QCs on the score plot (triangle labels) shows the stability of analytical conditions over different runs and therefore LC-MS system repeatability.

Accurate mass	VIP	P value
160.09642	7.69938	7.6E-11
338.08661	7.04354	1.1E-09
340.10223	5.99093	2.0E-15
144.06503	5.96467	3.5E-11
233.09152	5.91787	7.5E-11
210.07562	5.17122	1.6E-17
492.18539	5.04415	3.3E-04
195.0761	4.92066	6.4E-12
176.09137	4.89257	2.0E-10
247.10729	4.72567	4.3E-05
222.07558	4.46736	9.7E-07
146.0807	4.35058	6.3E-05
158.08078	4.03647	1.4E-04
224.09133	3.9584	8.0E-10
170.0323	3.87059	3.1E-10
176.09135	3.7477	4.2E-15
406.24319	3.73407	3.6E-10
146.05952	3.62707	7.5E-25
146.11717	3.56677	8.0E-10
255.06468	3.46605	2.2E-07
210.0605	3.39283	1.4E-04
251.10222	3.30837	3.7E-13
173.09161	3.20831	6.2E-07
212.20038	3.11975	3.7E-01
408.25876	3.1005	1.3E-06
132.06493	3.06205	1.1E-01
206.88556	2.9979	9.0E-10
285.07514	2.92111	1.8E-09
257.08029	2.8002	6.0E-08
133.06037	2.66863	6.5E-04
279.13359	2.62583	1.6E-05
438.26887	2.60702	1.0E-14
355.26269	2.59722	9.3E-04
260.14844	2.5621	3.6E-09
265.01575	2.5348	8.0E-03
121.07144	2.52708	2.0E-03
330.08148	2.51278	1.6E-05
251.10234	2.48931	4.6E-11
208.09644	2.48704	6.9E-06
149.08037	2.44043	3.5E-05
210.18491	2.3922	4.2E-04
231.07596	2.3363	2.4E-14
172.09639	2.33349	8.0E-08
334.16461	2.32748	2.8E-05
174.11214	2.29884	4.0E-11
382.20665	2.29279	1.5E-09
798.48413	2.28924	1.4E-11
348.1434	2.25969	2.1E-04
289.16412	2.21218	1.0E-04
261.09806	2.19099	3.8E-12
320.86584	2.15284	8.1E-09
392.1907	2.14263	1.0E-08
245.14914	2.11645	1.4E-08
208.06005	2.11177	5.3E-13
196.06002	2.0578	5.8E-05
224.05499	2.05012	5.5E-13

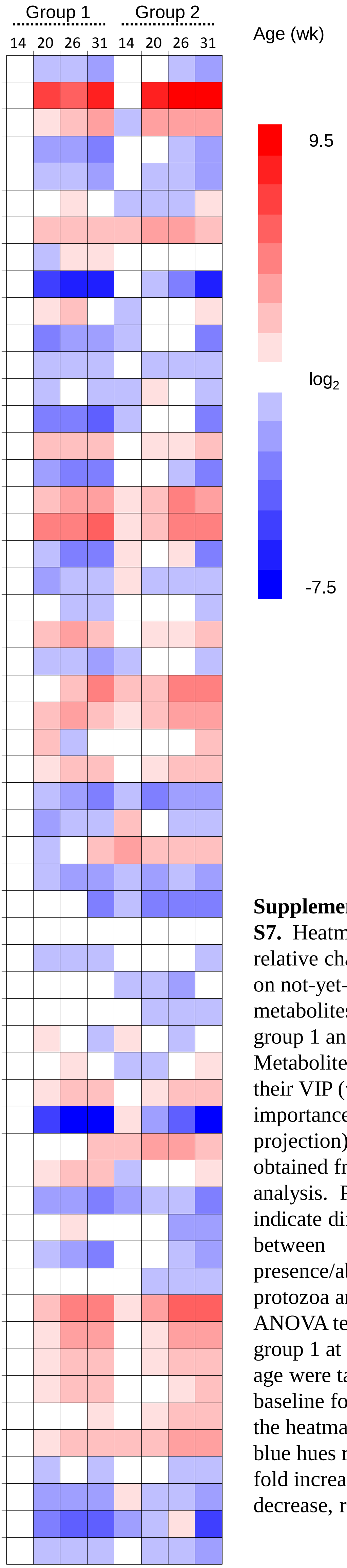

Supplementary figure S7. Heatmap showing relative changes in urine on not-yet-identified metabolites in lambs of group 1 and 2. Metabolites are ranged by their VIP (variable importance of the projection) value obtained from OPLS-DA analysis. P values indicate differences between presence/absence of protozoa analyzed by ANOVA test. Values for group 1 at 14 weeks of age were taken as the baseline for constructing the heatmap with red and blue hues representing fold increase and fold decrease, respectively.

2.2. Supplementary Tables

Supplementary Table S1. Rumen bacterial families in lambs as a function of interventions modulating the rumen microbiota[1]

Phylum	Family	Group	Age (week)												Comparison within group		
			14			20			26			31			14 vs 20	20 vs 26	26 vs 31
			mean	SE	*P*	mean	SE	*P*	mean	SE	*P*	mean	SE	*P*			
Bacteroidetes	Prevotellaceae	1	52.09	2.04		34.12	4.21		30.93	1.81	**	27.66	3.10		**		
		2	50.04	3.37		39.13	4.32		53.79	5.73		33.31	3.22			**	**
Bacteroidetes	[Paraprevotellaceae]	1	0.34	0.10		0.82	0.08	**	5.48	1.64	*	6.14	3.84		**	*	
		2	0.15	0.10		0.36	0.07		0.76	0.26		3.65	1.12				*
Firmicutes	Veillonellaceae	1	6.84	3.20		20.36	3.54	**	17.12	4.61	**	9.50	1.33		*		
		2	2.51	0.33		2.92	0.58		2.05	0.50		9.56	2.18				**
Firmicutes	Ruminococcaceae	1	18.39	1.66		4.82	1.74	*0.09*	3.79	0.74	**	3.44	0.75		**		
		2	13.52	5.48		13.84	4.81		12.52	0.98		3.84	0.71				**
Firmicutes	Lachnospiraceae	1	10.60	1.55		4.32	1.13		5.57	1.29		3.13	0.41		**		
		2	7.76	1.65		7.46	1.70		5.32	0.37		3.16	0.46				**
Firmicutes	Clostridiaceae	1	0.10	0.02		1.24	0.47	*	1.67	0.28	**	1.36	0.23		*		
		2	0.15	0.01		0.18	0.04		0.31	0.06		1.44	0.22				**
Firmicutes	[Coprobacillaceae]	1	2.65	1.02		0.39	0.16		1.18	0.55	*0.09*	0.20	0.13	*	*		
		2	5.65	2.32		1.47	0.77		0.21	0.05		0.94	0.32				**
Firmicutes	Erysipelotrichaceae	1	0.09	0.02		0.05	0.02	*0.07*	0.04	0.01		0.01	0.01				
		2	0.16	0.08		0.23	0.09		0.10	0.04		0.14	0.10				
Spirochaetes	Spirochaetaceae	1				0.30	0.11		0.247	0.055		0.12	0.03		*		
		2				0.12	0.02		0.122	0.056		0.15	0.02		**		
Tenericutes	Anaeroplasmataceae	1	0.02	0.02		0.57	0.20		1.01	0.51		0.66	0.11		*		
		2	0.14	0.13		0.42	0.21		0.21	0.12		2.49	1.46				

[1] At 15 weeks of age, lambs were gavaged with fresh (group 1) or freeze-thaw (group 2) rumen microbial inocula from adult sheep. From week 21 through 26 lambs received daily a mild dose of ochatoxin A, and from week 27 lambs were in a common pen. *= $P < 0.05$, **= $P < 0.01$. Trends ($P < 0.10$) are indicated with actual P values. Comparisons were made between groups for each period and within groups at different periods (n= 4).

Supplementary Table S2. Rumen archea OTUs in lambs as a function of interventions modulating the rumen microbiota[1]

Clade or genus	OTU	Group	Age (week)												Comparison within group		
			14			20			26			31			14 vs 20	20 vs 26	26 vs 31
			mean	SE	P	mean	SE	P	mean	SE	P	mean	SE	P			
Methanosphaera	8	1	20.68	10.29		3.22	3.20		0.000	0.000	*	0.09	0.05				0.05
		2	39.73	16.33		4.24	1.71		12.190	4.972		2.68	1.96		*		0.08
Methanosphaera	29	1	2.18	0.27	**	0.91	0.26		1.260	0.510		0.91	0.19		**		
		2	0.00	0.00		0.35	0.34		1.054	0.479		0.81	0.44				
Methanosphaera	98	1	1.12	0.72		0.66	0.49		0.199	0.199		0.02	0.02				
		2	0.11	0.11		0.23	0.22		0.170	0.170		0.05	0.05				
Methanosphaera	141	1	0.60	0.20		0.73	0.73		0.000	0.000	*	0.00	0.00				
		2	1.56	0.65		0.13	0.07		0.387	0.154		0.07	0.04		*		0.05
Gottschalskii clade	1	1	25.89	11.61	0.07	16.84	8.65		24.199	3.258	*	21.08	2.32				
		2	4.94	2.99		8.24	7.96		7.452	5.552		20.37	5.98				
Gottschalskii clade	41	1	4.38	1.90		4.47	2.02		6.209	0.556	**	3.54	0.61				*
		2	1.27	0.69		1.55	1.53		1.397	0.936		4.38	0.49				*
Gottschalskii clade	64	1	1.99	0.88	*	0.70	0.27		0.960	0.205	**	0.82	0.19				
		2	0.16	0.13		0.50	0.45		0.171	0.074		1.12	0.24				**
Gottschalskii clade	76	1	0.25	0.10		0.00	0.00		0.313	0.138		0.12	0.06	*	*	*	
		2	0.39	0.26		0.05	0.04		0.852	0.637		1.35	0.52				
Gottschalskii clade	101	1	3.68	1.29		11.05	6.99		10.411	4.895		10.49	4.78				
		2	1.22	1.08		3.50	3.46		2.996	1.895		10.56	2.75				*
Gottschalskii clade	158	1	1.11	0.59		0.33	0.11		0.469	0.137	0.08	0.51	0.16				
		2	0.19	0.12		0.19	0.19		0.146	0.112		0.36	0.07				
Gottschalskii clade	198	1	1.07	0.64		0.33	0.17		0.445	0.051	**	0.26	0.05				*
		2	0.05	0.05		0.24	0.24		0.076	0.043		0.20	0.06				0.09
Gottschalskii clade	106	1	0.00	0.00		0.02	0.02	0.09	0.696	0.356	0.07	0.37	0.15			0.07	
		2	0.00	0.00		0.00	0.00		0.000	0.000		0.89	0.52				0.09
Ruminantium clade	3	1	13.23	7.91		6.73	6.61		2.546	1.985	**	1.86	1.60				
		2	21.74	9.93		4.88	1.56		31.244	7.010		12.43	6.29			**	0.05
Ruminantium clade	30	1	2.02	1.33		0.71	0.70		0.577	0.487	**	0.41	0.37				
		2	5.01	2.95		0.88	0.30		5.644	1.296		2.26	1.25			**	0.07
Ruminantium clade	57	1	1.51	0.86		0.58	0.53		0.530	0.322		0.41	0.23				
		2	0.08	0.07		0.07	0.07		0.274	0.142		0.58	0.19				
Ruminantium clade	65	1	4.13	2.34		2.04	1.77		1.095	0.612		0.87	0.51				

		2	0.94	0.30		0.35	0.28		0.618	0.123		0.43	0.13				
Ruminantium clade	172	1	11.55	8.21		7.90	7.65		3.030	2.003		2.24	1.28				
		2	1.56	0.92		1.26	1.05		0.206	0.122		0.37	0.15				
Mb. boviskoriani/wolinii	6	1	0.00	0.00		0.66	0.44		14.831	6.046	*	12.57	3.83			*	
		2	0.05	0.05		0.31	0.31		1.142	0.418		11.17	6.32				
Mb. boviskoriani/wolinii	124	1	0.00	0.00		0.03	0.03		0.577	0.294		0.37	0.16			*0.09*	
		2	0.00	0.00		0.01	0.01		0.084	0.072		0.56	0.33				
Cand. Methanomethylophilus	2	1	0.00	0.00		7.22	7.20	*	0.000	0.000	*	0.13	0.07	*0.09*			*0.07*
		2	0.20	0.20		60.43	20.28		19.101	7.523		2.43	1.22		**	*	*
Cand. Methanomethylophilus	7	1	0.00	0.00		9.39	4.74	*0.07*	7.847	2.346	**	10.49	2.34		*0.07*		
		2	0.02	0.02		0.74	0.74		0.127	0.076		4.54	2.65				
Cand. Methanomethylophilus	9	1	0.00	0.00		3.41	3.41		10.680	6.883		7.69	2.89	*			
		2	0.00	0.00		0.12	0.12		8.997	8.661		0.91	0.56				
Cand. Methanomethylophilus	10	1	0.00	0.00		12.66	6.56		2.440	1.158	*0.08*	5.53	2.62		*0.08*		
		2	0.00	0.00		3.22	3.22		0.279	0.279		6.20	3.55				
Cand. Methanomethylophilus	11	1	1.67	1.49		3.49	1.31	*0.08*	4.143	2.370		6.57	3.95				
		2	1.12	1.12		0.80	0.80		0.549	0.373		6.70	2.53				*
Cand. Methanomethylophilus	18	1	0.00	0.00		1.39	0.64		0.827	0.398		3.13	1.70				
		2	0.44	0.44		1.83	1.83		0.201	0.106		0.73	0.26				*0.07*
Cand. Methanomethylophilus	34	1	0.00	0.00		0.41	0.29		0.100	0.072		1.00	0.32				*
		2	0.00	0.00		0.01	0.01		0.292	0.180		0.46	0.20				
Cand. Methanomethylophilus	55	1	0.13	0.13		0.31	0.11	*0.09*	0.465	0.247		0.88	0.47				
		2	0.13	0.13		0.07	0.07		0.060	0.060		0.90	0.53				
Cand. Methanomethylophilus	179	1	0.00	0.00		0.79	0.49		0.174	0.066		2.84	1.66				
		2	0.00	0.00		0.00	0.00		0.297	0.182		0.56	0.43				
Cand. Methanomethylophilus	192	1	0.00	0.00		0.07	0.07	*	0.000	0.000	*0.05*	0.00	0.00				
		2	0.02	0.02		1.71	0.58		0.559	0.263		0.11	0.06		**	*0.06*	

[1] At 15 weeks of age, lambs were gavaged with fresh (group 1) or freeze-thaw (group 2) rumen microbial inocula from adult sheep. From week 21 through 26 lambs received daily a mild dose of ochatoxin A, and from week 27 lambs were in a common pen. *= $P < 0.05$, **= $P < 0.01$. Trends ($P < 0.10$) are indicated with actual P values. Comparisons were made between groups for each period and within groups at different periods (n= 4).
